# The Anticoccidial Effect of Alcoholic *Vitis vinifera* Leaf Extracts on *Eimeria papillate* Oocysts Isolated in Mice In Vitro and In Vivo

**DOI:** 10.3390/vetsci10020097

**Published:** 2023-01-29

**Authors:** Mutee Murshed, Saleh Al-Quraishy, Jawahir Alghamdi, Hossam M. A. Aljawdah, Mohammed M. Mares

**Affiliations:** Department of Zoology, College of Science, King Saud University, P.O. Box 2455, Riyadh 11451, Saudi Arabia

**Keywords:** *Vitis vinifera* extract, eimeriosis, oocysts, sporulation, in vitro, mice

## Abstract

**Simple Summary:**

Coccidiosis is a disease affecting an enormous group of animals such as birds and mammals. It is caused by an *Eimeria* spp. infection, which induces gastrointestinal issues that chiefly affect the intestines such as diarrhea, reduced growth performance, weight loss, and reduced feed conversion efficiency; in extreme cases, it can lead to death. In addition, this disease causes substantial economic losses globally. The extensive use of the currently obtainable anticoccidial treatments has led to expansive medication resistance, prompting the investigation of new treatments. *Vitis vinifera* leaf extract is an interesting potential candidate for an alternative treatment approach. Our study examined the pharmacological potential of VVLE and its effects on *E. papillata* oocyst sporulation by investigating its ability to inhibit the sporulation of coccidian oocysts in vitro and in mice.

**Abstract:**

*Eimeria* spp. causes eimeriosis in the guts of numerous domestic mammals and poultry, and the employment of medication and the effects of certain aspects of synthetic anticoccidials in the treatment of eimeriosis have given rise to the appearance of resistant parasites that require the search for alternate remedies. Natural products, which are safe and have no negative impact on the environment, may be utilized in the therapy of an enormous range of parasitic infections. This research aimed to assess the effectiveness of VVLE on the oocyst sporulation of an *E. papillate* infection in the mouse jejunum. In addition, obtaining the ideal concentration will interrupt the parasite’s life cycle and limit infection. In vitro: Collected unsporulated oocysts (1 × 10^3^) of *E. papillata* were given six different concentrations (*w*/*v*) of *Vitis vinifera* leaf extract (10, 25, 50, 100, 150, and 200 mg/mL) and toltrazuril (25 mg/mL), three replicates per group, whereas the control group received 2.5% potassium dichromate solution. In vivo: The mice were separated into six groups; the first and second groups did not receive infection, whilst the third, fourth, fifth, and sixth groups were each given 1 × 10^3^ sporulated oocysts of *E. papillate* in the experiment. In addition, an oral dosage of 100 and 200 mg/kg VVLE were given to the fourth and fifth groups, while the sixth group was given toltrazuril at 25 mg/kg. On the fifth day, unpopulated oocysts were collected from each mouse separately. The incubation period and treatments had considerable impacts on the rate of sporulation. The infrared spectroscopy of *V. vinifera* extract revealed many expected active classes of chemical compounds. Further, the infection of mice with *E. papillata* caused an oocyst output of nearly 2 × 10^4^ oocysts/g of faeces. VVLE significantly decreased the oocyst output to nearly 88%. In addition, we detected an inhibitory effect on the sporulation (%) and harm (%) of *E. papillata* oocysts in a dosage-dependent modality compared with the control group. Furthermore, they destroyed the oocyst morphology in terms of the shape, size, and quantity of sporocysts. The results indicate that grape vines have powerful activity as anticoccidials.

## 1. Introduction

Coccidiosis is a dangerous disease that affects a wide range of animals [1]. It is caused by *Eimeria* spp. infection, which invades host cells in the infectious stages and causes gastrointestinal issues such as diarrhea, leads to decreased growth performance, and, in extreme cases, leads to death [2,3]. Moreover, *Eimeria* spp. infections can lead to opportunistic infections of other pathogens such as bacteria [4]. In addition, this disease causes substantial economic losses globally [5]. It has a life cycle that consists of asexual and sexual stages of reproduction, and it produces resistant parasite stages known as oocysts that are released into the environment, thus facilitating the spread of infection. Controlling the *Eimeria* oocysts is difficult due to their relative resistance to environmental factors [6]. As an outcome, deactivating the sporulation operation is an essential step by which these parasites can be controlled [7]. Furthermore, the extensive prophylactic usage of anticoccidial-added feed has led to extensive medication resistance [5], which has presently been reported against attainable drugs [4,8], e.g., amprolium, sulfonamides, diclazuril, halofuginone, decoquinate, clopidol, and toltrazuril; along with the difficulty of finding an accessible and scalable anticoccidial vaccine, it may be costly and inaccessible to rural farmers and others in the poultry industry [8,9]. An increased interest in safe and effective alternatives aimed at controlling coccidiosis has led to the use of plant extracts, essential oils, and traditional medicinal products [10]. Medicinal plants are the primary source of all alternative or natural medical systems, and they are regarded as therapeutic approaches for obtaining effective chemical ingredients [11]. These products exhibit organ-protective characteristics in Eimeria-infected hosts and target parasites [12].

Lately, some emphasis has been placed on herbal therapy. Plants such as *Pinus radiata* and *Aloe vera* [13], *Saccharum officinarum* [14], *Rumex nervosus* [15], *Cinnamomum Verum* [16], and *Calotropis Procera* [17] were reported as having good antiparasitic and immunomodulatory effects against coccidiosis [18]. Several reviews have described the phytochemical and therapeutic effects of grape (*Vitis vinifera*) and the active constituents in different parts of the fruit, including the skin, seeds, pomace, and stems [19]. Its primary ingredients have a variety of bioactivities, such as cardioprotection, anticancer, anti-inflammation, antiaging, and antimicrobial activities, which are closely related to disease prevention [20]. In addition to these possible efficacies, it has as an antiparasitic agent [21,22].

Grape leaves contain many compounds with antioxidant activity, such as polyphenols, which include monomeric flavonoids (catechin and epicatechin), tannic acid, ellagitannin, dimeric, trimeric, and polymeric procyanidins, and phenolic acids such as ellagic and Gallic acid [23]. Phenolic compounds and the useful influences from *V. vinifera* leaves contribute directly to the protective effect observed in the brains of mice, in addition to lowering lipid and protein damage and modifying the status of the antioxidant enzymes investigated [24].

The *V. vinifera* leaves have been utilized in the treatment of a wide variety of diseases such as in the treatment of hypertension, diarrhea, hemorrhage, varicose veins [21,22], and inflammatory turmoil [25], and to lower blood glucose levels [26]. The leaves of *V. vinifera* have also been shown to be hepatoprotective against acetaminophen-induced hepatic DNA damage [27].

The present investigation was carried out to assess the pharmacological potential of grape leaves and their effects on *E. papillata* oocyst sporulation and viability in vitro and in infected mice.

## 2. Materials and Methods

Thirty-five mice were bought from animal markets. The mice were screened for seven days before the start of the experiment to confirm that they were free of Eimeria infection through fecal investigation, and no *Eimeria* oocysts were obtained.

### 2.1. Ethical Approval 

The research was carried out in accordance with the ethical criteria for the use of animals established by the Kingdom of Saudi Arabia (Ethic Committee King Saud University, ethical approval number: KSU-SE-21-86).

### 2.2. Preparation of Extract

The preparation of the grape leaf extract followed the procedure that was outlined by [28], using fresh leaves collected from Shaqra, Saudi Arabia, and a classification scientist at King Saud University’s Botany Department validated the plant’s botanical identity. The air-dried leaves (200 g) were ground into a powder and then extracted by percolating with 70% methanol at room temperature and then conserved for 24 h at 4 °C with occasional stirring. The resulting extract was then concentrated and dried using a rotating vacuum evaporator (Yamato RE300, Tokyo, Japan) under reduced pressure at 40 °C. The obtained crude extracts were weighed and stored at (−20 °C) until used for experiment purposes. A solution of potassium dichromate (K_2_Cr_2_O_7_) and distilled water were employed to dissolve the powder for the different trials.

### 2.3. Infrared Spectroscopy

After processing, a tiny piece of the material was homogenized by combining it with an excessive amount of potassium bromide powder (1:99 wt%). The material was then given a rough mashing before being put in a pellet-forming die. The optical spectrometer NICOLET 6700 Fourier-transform infrared spectroscopy from Thermo Scientific was used to examine infrared (IR) (FT-IR) to predict the likely compound classes. The maximum absorption is expressed as the number of waves (cm^−1^). At 25 °C, spectra with a resolution of 4 cm were recorded, spanning from 4000 cm^−1^ to 400.

### 2.4. Oocyst Sporulation

Unpopulated *Eimeria papillata* oocysts were found by Prof. Mehlhorn, University of Duesseldorf (Duesseldorf, Germany), and the parasite was kept alive by the passage in mice [29]. The treatment was used in vitro through oocysts and was investigated using dichromate. In vivo, sporulation was performed prior to giving it to the mice in order to activate and propagate parasite oocysts.

### 2.5. In Vitro

A suspension of 654 µL of freshly sporulated oocysts of *E. papillata* containing 1 × 10^3^ oocysts was added to each six-well plate and then these dishes were incubated in 3 mL of potassium dichromate containing one of the following concentrations of VVLE: 10%, 25%, 50%, 100%, 150%, or 200%. The potassium dichromate solution (K_2_Cr_2_O_7_) was only used in the negative control group, while toltrazuril (Veterinary Agriculture Products Company (VAPCO)) was used as a positive control group (eight groups). Three replicates were employed for each concentration. All six-well plates for all groups were incubated at 25–29 °C and 65–75% relative humidity and all were semi-covered, with periodic shaking [27]. From each group, a 10 µL suspension was examined microscopically under a light microscope (BX51TF, OLYMPUS, Tokyo, Japan) at 40× magnification, after 24, 48, 72, and 96 h. The viability (oocysts with sporocysts, distorted walls, and inhibitory oocysts) was examined under the light microscope using a McMaster chamber, and the percentages of sporocysts and inhibitory oocysts were calculated using the following equations:

Sporulation (%) = number of sporulated oocysts/total number of oocysts × 100.

The inhibition of sporulation (SI%) = Sporulation of control-sporulation of treated/sporulation of control × 100 as described by [28].

### 2.6. In Vivo

In this study, adult male C57BL/6 mice aged 10–11 weeks with an average weight of 26–27 g/mice were subjected to the experiment. The mice were divided into five groups with seven mice in each group. The first group was inoculated only with sterile saline (0.9% NaCl) and served as the control group. The second group (noninfected) was treated by oral gavage with 100 μL *V. vinifera* leaf extract (200 mg/kg) daily for 5 days. The third, fourth, and fifth groups were infected with 1 × 10^3^ sporulated oocysts of *E. papillata* and treated either with sterile saline, *V. vinifera* leaf extract, or 25 mg/kg toltrazuril daily for 5 days, respectively.

### 2.7. Sample Collection

Fresh fecal pellets were collected every 24 h and weighed for all mice, and the bedding of the mice was changed to prevent reinfection. On day five after infection, each mouse was removed to a small cage, fresh fecal samples were collected from the mice, and the number of oocysts shed per 1 g of feces was calculated according to [30]. The feces pellets from each individual mouse were suspended and diluted in saturated sodium chloride (NaCl) solution for oocyst flotation. The oocysts were counted using a McMaster chamber, and the upshots are expressed as the number of oocysts per gram of feces. To assess the mice’s weight change, each mouse was weighed separately before starting the experiment and again at 5 days post-infection.

### 2.8. Statistical Analysis

The SPSS software (IBM SPSS, Version 23) was used. Tukey’s method and variables were analyzed using the following experimental design through a two-way ANOVA model for in vitro study:

Y_ijk_ = μ + G_i_ + T_j_ + GT_ij_ + ε_ijk_

where Y_ijk_ is the value of any observation, μ is the population mean of the measurements, Gi is the effect of the ith treatment groups, T_j_ is the effect of the jth time of incubation, GT_ij_ is the effect of the ijth interaction between treatment and incubation time, and ε_ij_ is random error.

In vivo study model:

Y_ij_ = μ + T_i_ + ε_ij_

where Y_ij_ is the value of any observation, μ is the population mean of the measurements, T_i_ is the effect the ith treatment groups, and ε_ij_ is random error.

In order to evaluate the overall effects of the treatment in vitro and in vivo, comparisons were made between all groups.

The arithmetic mean (M) and standard deviation (SD) were used to represent the results. Statistically significant differences (*p* ≤ 0.05) were discovered between treatment groups and control groups.

## 3. Results

**Figure 1:**
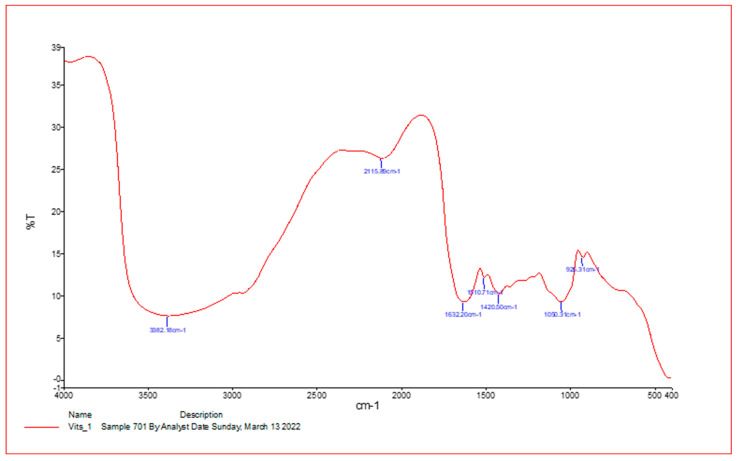
The results of undertaking infrared spectroscopy on VVLE samples. An FT-IR spectrometer was used to collect the data, and the findings were collected in the range of 400–4000/cm^−1^.

An FT-IR spectrometer was used to perform spectroscopy on the VVLE, analyzed with major bands at 3382.18 cm^−1^, 2119.89 cm^−1^, 1632.20 cm^−1^, 1510.71 cm^−1^, 1420.50 cm^−1^, and 1030.31 cm^−1^, respectively, as shown in Figure 1. The active phytochemical components were obtained as follows: N-H stretching, N=C=S stretchy, C=C stretching, C-O stretching tertiary, CO-O-CO stretching, and C-H bending, with an absorbance of 400–4000/cm^−1^ (Table 1).

The proportion of sporulated oocysts was calculated for the control and experimental groups at 24, 48, 72, and 96 h. It is important to note that oocysts started to sporulate after 48 h of incubation. The lowest sporulated oocyst percentages were noted in concentrations of 10, 25, and 50 mg/mL. The concentrations of the grape leaf extracts were inversely proportional to the percentage of sporulated oocysts.

**Figure 2:**
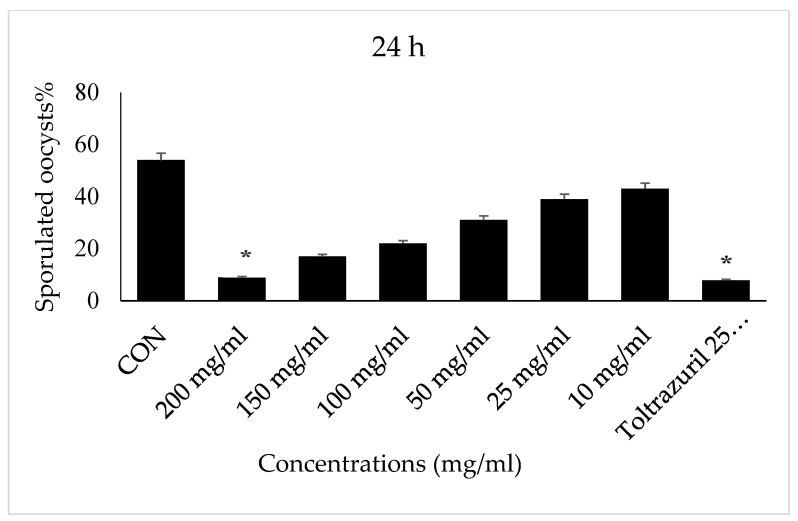
Effect of VVLE on the sporulation of *E. papillata* oocysts at 48 h in vitro. The significance was determined by comparing it to a negative control consisting of potassium dichromate 2.5% and a positive control consisting of toltrazuril 25 mg/mL. Significance is in relation to the group that served as a control (*p* < 0.05). Significance (*): *p*-value ≤ 0.05. (VVLE): *Vitis vinifera* leaf extract. (h): hours.

After 24 h of *E. papillata* oocyst incubation in concentrations of 200 mg/mL with VVLE, and toltrazuril 25 mg/mL, there was no sporulation. However, a concentration of 150 mg/mL showed less than 5% sporulation. Concentrations of 10, 25, 50, 100, and 150 mg/mL, showed different levels of sporulation. While the control group (2.5% potassium dichromate solution) remained unsporulated throughout the incubation time until 96 h (Figure 2), we note no significant differences between treatments with all concentrations of VVLE and drug and potassium dichromate (2.5%).

After 48 h of incubation with VVLE (200 µg/mL and toltrazuril 25 mg/mL), the *E. papillate* oocysts showed high levels of inhibition in comparison with the 2.5% potassium dichromate (control), which showed high levels of sporulated oocysts, while oocysts incubated with VVLE (150,100, 50, 25, and 10 mg/mL) showed low levels of sporulation.

**Figure 3:**
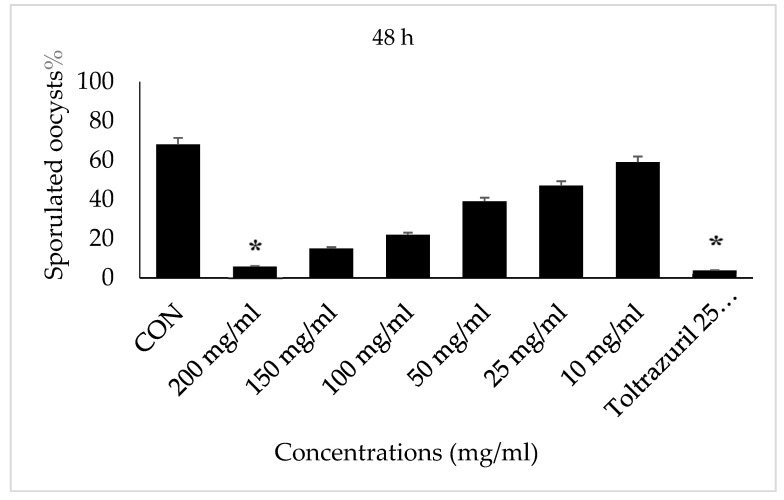
Effect of VVLE on sporulation of *E. papillata* oocysts at 48 h in vitro. The significance was compared to potassium dichromate 2.5% as a negative control and toltrazuril 25 mg/mL as a positive control. Significance is in comparison to the control group (*p* < 0.05). Significance (*): *p*-value ≤ 0.05. (VVLE): *Vitis vinifera* leaf extract. (h): hours.

**Figure 4:**
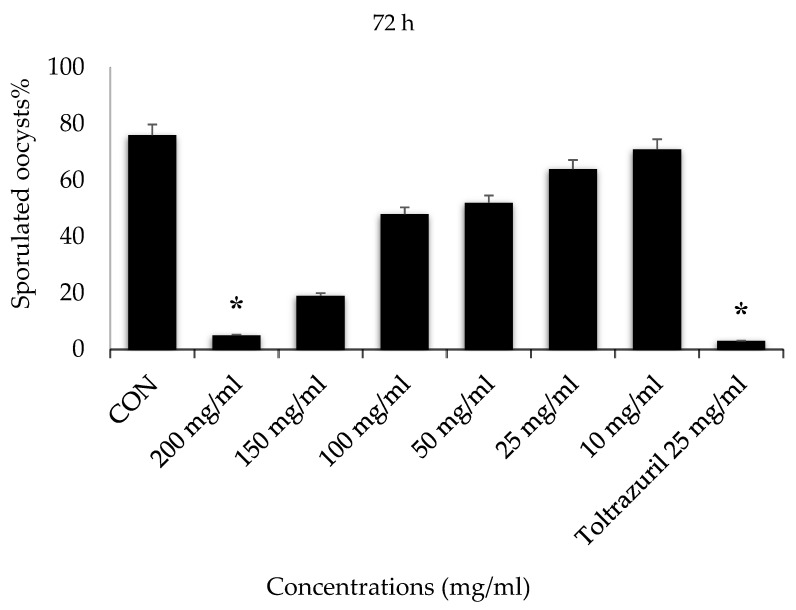
Effect of VVLE on sporulation of *E. papillata* oocysts at 72 h in vitro. The significance was determined by comparing it to a negative control consisting of potassium dichromate 2.5% and a positive control consisting of toltrazuril 25 mg/mL. Significance is in relation to the group that served as a control (*p* < 0.05). Significance (*): *p*-value ≤ 0.05. (VVLE): Vitis vinifera leaf extract. (h): hours.

Incubation for 72 h with VVLE at concentrations of 200 mg/mL and toltrazuril inhibited sporulation by nearly 70–80%, whilst incubation with VVLE at the same concentration and toltrazuril for 96 h inhibited sporulation by nearly 88% compared to the control group in which oocysts were incubated with potassium dichromate (2.5%). A reduction in the population of parasites was also obtained at various concentrations throughout 72–96 h for the extract compared with toltrazuril (Figure 3 and Figure 4).

**Figure 5:**
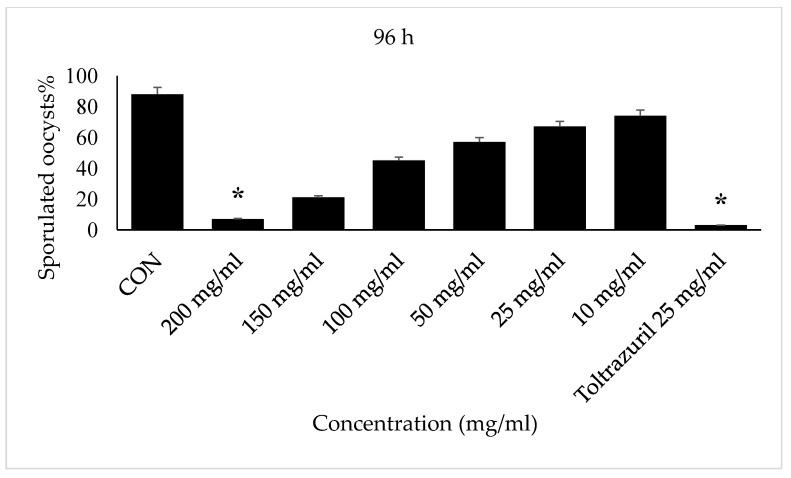
Effect of VVLE on sporulation of *E. papillata* oocysts at 96 h in vitro. The significance was determined by comparing it to a negative control consisting of potassium dichromate 2.5% and a positive control consisting of toltrazuril 25 mg/mL. When compared to the control group, significance was found (*p* < 0.05). Significance (*): *p*-value ≤ 0.05. (VVLE): Vitis vinifera leaf extract. (h): hours.

**Figure 6:**
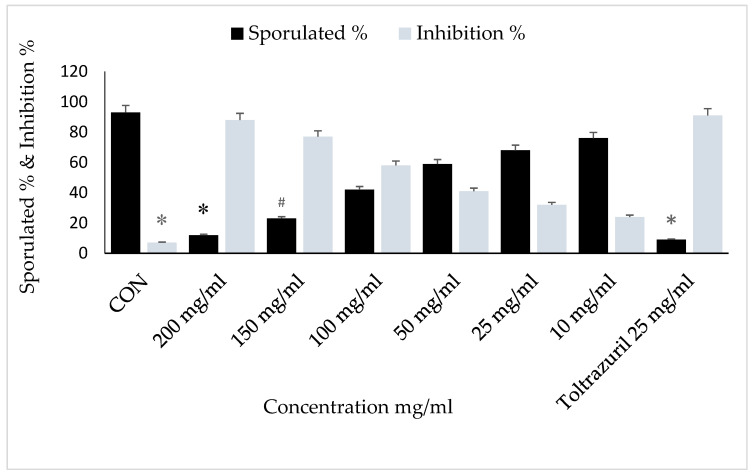
In vitro main contact time effects of VVLE on sporulation %, inhibition %, of *E. papillata* oocysts at different times for all concentrations. The significance was determined by comparing it to a negative control consisting of potassium dichromate 2.5% and a positive control consisting of toltrazuril 25 mg/mL. Significance is in comparison to the control group. Significance (*): *p*-value ≤ 0.01. (#) *p*-value ≤ 0.05. (VVLE): *Vitis vinifera* leaf extract. (h): hours.

**Figure 7:**
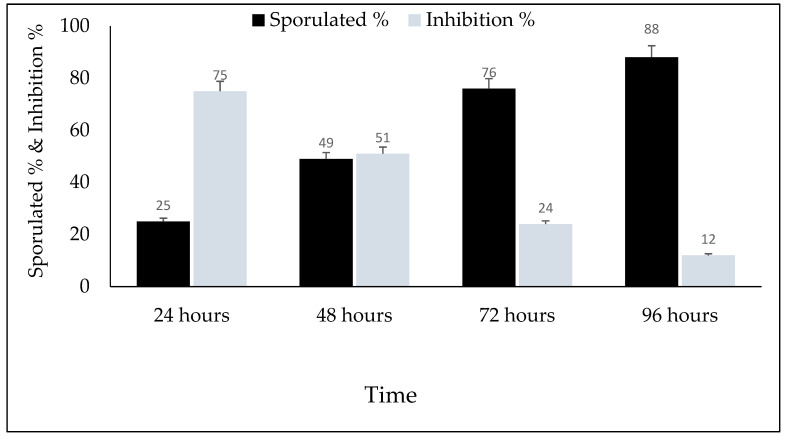
In vitro main effects of VVLE on sporulation %, inhibition%, of *E. papillata* oocysts at different concentrations during 24, 48, 72, and 96 h. The significance was compared to potassium dichromate 2.5% as a negative control and toltrazuril 25 mg/mL as a positive control. (VVLE): Vitis vinifera leaf extract. (h): hours.

The main effects of the time of sporulation and experimental groups on sporulation (%) and the sporulation inhibition (%) of *E. papillata* oocysts are shown in Figure 5, which shows that the sporulation percentage increased with an increasing incubation time; conversely, as shown in Figure 6 and Figure 7, the inhibition percentage increased significantly with an increasing incubation time of up to 72 h (*p* ≤ 0.05). Therefore, the sporulation inhibition rate did not differ significantly between 72 and 96 h exposures. There were interactions between the timing of sporulation and the experimental groups on the sporulation and inhibition of *E. papillata* oocysts.

The influence of grape leaf extract on the outcome of *E. papillata* infections was studied. There was no fecal output of oocysts during the first 4 days post-infection. On day five post-infection, the highest oocyst output was 8.5 × 10^5^ ± 29.7 oocysts/g of feces in infected mice. While the grape treatment significantly reduced oocyst output to about 65% on day five post-infection (Table 2), the treatment VVLE at doses of 100, 150, and 200 mg/kg significantly (*p* < 0.01) reduced the oocyst output to 78.8%, 86.5%, and 96.4%, respectively (Figure 5).

**Figure 8:**
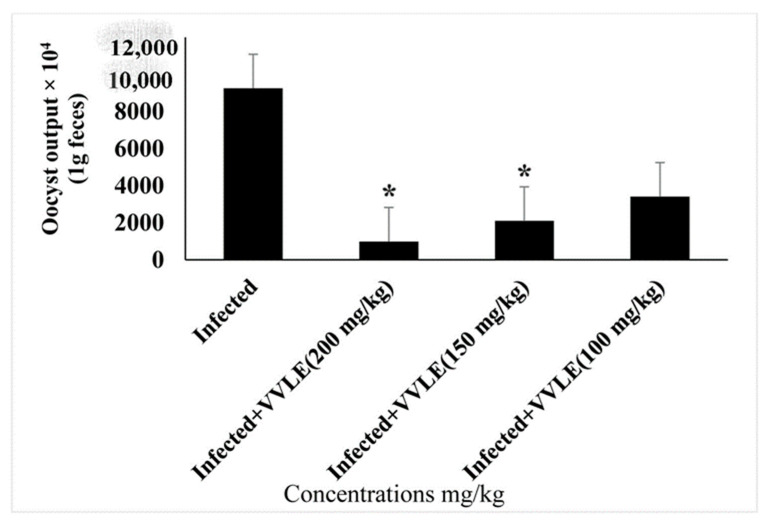
The effect of different VVLE doses on oocyst output on day five after *E. papillata* oocyst infection. * Significance is in comparison to the infected group (*p* ≤ 0.05).

**Figure 9:**
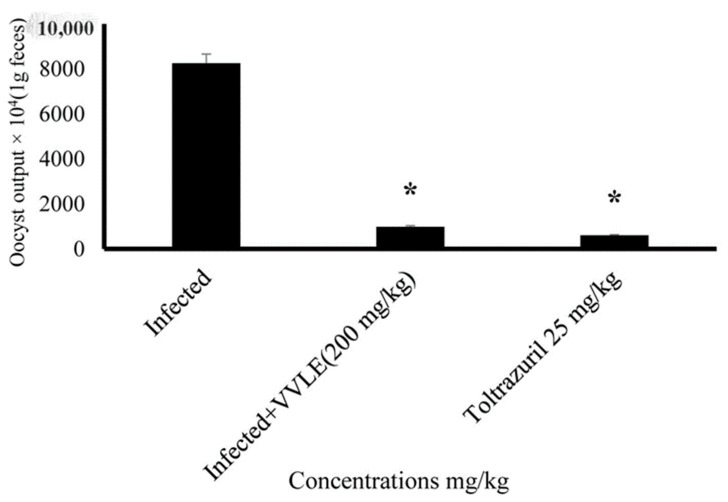
Sporulation oocysts count in feces of infected, VVLE, and toltrazuril-treated mice. *, the difference between the infected treated groups is significant at *p* ≤ 0.05.

Generally, the experimental groups significantly affected the rates of sporulation (%) and inhibition (%). Concentrations of 200, 150, and 100 mg/mL of VVLE had the highest inhibition rates and lowest sporulation rates (*p* ≤ 0.05). Concentrations of 50, 25, and 10 mg/mL of VVLE had higher rates of sporulation and the lowest inhibition of sporulation (*p* ≤ 0.05) (Figure 8 and Figure 9).

## 4. Discussion

The in vitro and in vivo anticoccidial effects of *Vitis vinifera* extracts were measured in terms of the number and percentage of Eimeria oocysts damaged and sporulation inhibition. Eimeriosis has caused great economic losses in many countries as it affects most animals [31]. When sporozoites from swallowed sporulated oocysts are released, they enter the jejunum epithelial cells and rapidly multiply, causing *E. papillata* infections before oocysts are produced. Progeny occurs within the oocysts outside of the host, and they become contagious [32]. In many studies, Eimeriosis drug resistance has been reported [5,33]. Therefore, attention is being paid to using natural products as antiparasitic agents since they are more effective, less toxic, and have fewer adverse effects than conventional chemical agents [12]. Several plant extracts have been used in recent years for the control and treatment of infectious disorders in poultry animals, such as coccidiosis. Many findings reveal that the methanolic extract from the grape plant has anticoccidial properties [34,35]. Our findings revealed a dose-dependent inhibitory effect on Eimeria oocyst sporulation and damage. *V. vinifera* extracts were utilized to treat mice with experimentally induced coccidiosis. It possessed many several active chemical classes that could be conceivably expected.

This corroborates the findings of several authors who claimed that some plants, such as *Saccharum officinarum*, had anticoccidial properties [36,37,38], including *Pinus radiata* [39] and *Aloe vera* [40,41]. Additionally, oocysts showed various degrees of damage, including cell wall damage, and sometimes the oocyst wall completely disappeared and only free sporocysts were seen [42]. This harm may be caused by anaerobic conditions induced by the grape crude leaf extract. This could be because of the active components in the leaf extract. Anaerobic digestion, which is made up of a sequence of microbial processes, turns the waste’s organic content into biogas and has a detrimental impact on pathogens. These were compared to controls, which were normal *E. papillata* oocysts at various stages. A reduction in the parasite population was also observed at various doses of the plant extract over a 96-h period compared to toltrazuril and the control.

We found that VVLE, at a concentration of 200 mg/mL, had an influence on oocyst sporulation. This effect may be attributed to the existence of several bioactive phytochemical ingredients [43]. VVLE showed anti-coccidiosis efficacy following the therapy of mice, as confirmed by a significant decrease in the output of *E. papillata* oocysts in the faeces and oocysts of infected mice. These results agree with those of other studies that investigated *Azadirachta indica* [44], *Punica granatum* plant [45], *Ziziphus Spina*-Christi extract [46], Mulberry extract [47], and grape [42] extract as possible sources of anticoccidial agents, as well as *Calotropis procera* leaf extract [17].

Various herbal extracts are commonly employed in chicken diets to increase growth rates and animal health, especially when health difficulties are present. Several studies have found that plant extracts improve poultry performance, diet, and conversion efficiency [48,49]. Since VVLE has an anticoccidial action, it could be possible to employ it as a food additive in chicken feed.

Similar in vitro results were reported by [13], who found the sporulation of Eimeria oocysts was inhibited by aqueous pine bark extract. The in vitro anticoccidial efficacy of the ethnomedicinal plants was found to be concentration-dependent.

This gives rise to the possibility that the impact is pharmacologically mediated, demonstrating that the concentrations of the bioactive components may be higher in the plant extracts that have higher dilutions (i.e., 200 and 150 mg/kg). Similar results were obtained by [50], and it also was found that *Aloe vera* and *Thonningia sanguine* extracts showed concentration-dependent effects on the oocysts of *E. tenella*, and *P. biglobosa* extracts had lower anticoccidial effectiveness [51,52].

This might be a result of the lack of bioactive substances in them or of low levels of such compounds. In contradiction to what was stated in [53], there was in fact anticoccidial action seen in vitro of *K. senegalensis*, the current investigation found that the plant extracts have anticoccidial activity against *E. papillata* oocysts. This may be an outcome of the permeation of the cell walls of the oocysts by the fundamental plant components causing damage to the cytoplasm, which was evidenced by the manifestation of abnormal sporocysts in oocysts exposed to 200 and 150 dilutions in most of the plant extracts [42].

Our findings show that grape leaves have potent antiparasitic and anti-sporulation leverage. More research is required to understand the histological and molecular mechanisms of *Vitis vinifera* leaf extract sporulation suppression and protective effects against *E. papillata*-induced small intestine damage.

## 5. Conclusions

Experiments in vitro and in vivo have shown that the extracts have strong effects on the prevention of oocyst sporulation. More extensive pharmacological research should be conducted to determine the new pharmacodynamic effects of bioactive components. Additionally, the primary mechanisms underlying the bioactivities should be investigated in a comprehensive manner. It is necessary to clarify the histological and molecular mechanisms of sporulation inhibition by *Vitis vinifera* (grape) and its protective influence against *E. papillata*-induced intestinal contagion.

## Figures and Tables

**Table 1:** IR spectrum of *Vitis vinifera* leaf extracts by frequency range.

Absorption (cm^−1^)	Appearance	Transmittance (%)	Group	Compound Class
3425.53	medium	12	N-H stretching	Aliphatic primary amine
2093.10	strong	47	N=C=S stretchy	Isothiocyanate
1641.41	strong	25	C=C stretching	Alkene
1209.12	strong	37	C-O stretching tertiary	alcohol
1045.64	Strong broad	35	CO-O-CO stretching	Anhydride
410.42	strong	3	C-H bending	1,2-disubstituted

**Table 2:** Effect of VVLE on oocyst output on day five post-infection with *E. papillata* oocysts.

	Weight of Mice (g)		Oocyst Output/g Feces
	Day 0	Day 5	
Noninfected (−vitis)	28.19 ± 1.4	28.70 ± 1.4	0
Noninfected (+vitis)	26.9 ± 1.16	27.06 ± 0.98	0
Infected (−vitis)	26.5 ± 1.4	26.3 ± 1.1	828.75 ± 54.668
Infected (+vitis)	26.51 ± 0.74	26.83 ± 0.746	28.808 ± 6.785 *
Toltrazuril 25 mg/mL	26.17 ± 1.15	26.98 ± 1.5	26.743 ± 6.321 *

* Significance against the infected group (*p* < 0.01).

## Data Availability

Not applicable.
